# High-frequency ultrasound-derived imaging biomarkers in inflammatory skin diseases

**DOI:** 10.3389/fmed.2026.1972814

**Published:** 2026-10-14

**Authors:** Claudia Gonzalez, Cesar Gonzalez, Carolina Palacio, Laura Trujillo, Carlos Bravo, Lucia Achell, George Kroumpouzos

**Affiliations:** 1 Highly Specialized Ultrasound Center, Bogotá, Colombia; 2 Dermatology Practice, Bogotá, Colombia; 3 Hair and Skin Evolution Practice, Bogotá, Colombia; 4 Dermatologia Integral, San José, Costa Rica; 5 Dermatology Department, Centro Médico Nacional “20 de Noviembre” ISSSTE, Mexico City, Mexico; 6 Department of Dermatology, Alpert Medical School at Brown University, Providence, RI, United States; 7 APDerm, South Weymouth, MA, United States

**Keywords:** dermatologic ultrasound, Doppler ultrasound, elastography, high-frequency ultrasound, imaging biomarkers, inflammatory skin diseases, precision dermatology, treatment monitoring

## Abstract

**Background:**

Inflammatory skin diseases produce structural, vascular, and tissue-remodeling changes that may extend beyond abnormalities detectable by clinical examination. High-frequency ultrasound (HFUS), including ultra-high-frequency systems, provides noninvasive, real-time assessment of cutaneous morphology and inflammatory vascular activity.

**Objective:**

To examine HFUS in inflammatory skin diseases from an imaging biomarker perspective, emphasizing measurable structural, vascular, and mechanical features and their potential clinical applications.

**Key findings:**

Candidate ultrasound biomarkers include epidermal and dermal thickness, echogenicity, tissue interfaces, perifollicular abnormalities, fluid collections, fibrosis, architectural distortion, Doppler-detected vascularity, and tissue stiffness. Their clinical relevance is particularly evident in psoriasis, atopic dermatitis, acne vulgaris, hidradenitis suppurativa, and morphea, with emerging applications in other inflammatory dermatoses. In selected disorders, HFUS may reveal clinically occult abnormalities, while treatment-associated changes in selected parameters support their potential as candidate treatment-response biomarkers. Quantitative image analysis, elastography, and artificial intelligence may further improve reproducibility and interpretation.

**Conclusions:**

HFUS is evolving from predominantly morphological imaging toward a platform for deriving imaging biomarkers in inflammatory skin disease. Broader clinical implementation will require standardized acquisition, validated biomarkers and thresholds, operator training, and prospective evidence demonstrating clinical utility.

## Introduction

Inflammatory skin diseases are a major source of chronic morbidity worldwide (1). Although biologically diverse, many produce structural and vascular alterations that extend beyond clinically visible changes (2). Objective assessment of these abnormalities is increasingly relevant to evaluating disease activity, tissue damage, and treatment response. Clinical examination, severity scores, and histopathology remain fundamental but have limitations: clinical assessment may underestimate deeper inflammation, whereas biopsy is invasive, samples a limited area, and is poorly suited to serial monitoring (3). These limitations have increased interest in noninvasive imaging techniques that enable real-time assessment of cutaneous and subcutaneous structures.

High-frequency ultrasound (HFUS) permits detailed evaluation of the epidermis, dermis, superficial subcutaneous tissue, and appendages, while Doppler techniques provide complementary information on vascular activity (4–7). Higher-frequency systems provide greater spatial resolution of superficial structures at the expense of penetration depth (8, 9). Terminology regarding frequency ranges is not fully standardized. The EFSUMB position statement recommends a minimum frequency of 15 MHz for dermatologic ultrasound and describes transducers >20 MHz as very-high frequency and 30–70 MHz as ultra-high frequency (10). More recently, the WFUMB position paper similarly recommends linear or compact linear multifrequency transducers with an upper frequency range of at least 15 MHz as a minimum technical requirement and illustrates the substantially greater spatial resolution achievable at 70 MHz compared with 15 MHz (11). Other contemporary dermatologic ultrasound literature uses somewhat different operational ranges, including 20–30 MHz for HFUS and approximately 48–70 MHz for UHFUS (12). Increasing frequency primarily improves visualization and delineation of small superficial structures, rather than inherently increasing the numerical accuracy of millimetric measurements once the structure is adequately resolved. For quantitative assessment, comparability across frequencies and platforms depends on adequate structural visualization, calibration, acquisition technique, image quality, and standardized measurement methodology. This consideration is particularly relevant to epidermal thickness, because quantitative measurement is reliable only when the epidermal entry echo is clearly resolved; caution is therefore required when extrapolating measurements obtained at lower frequencies to thin normal epidermis or across disease states. In this review, HFUS is used as a general term when discussing dermatologic ultrasound collectively, whereas UHFUS is retained when used in the original study or when frequency-specific differences materially affect image resolution or quantitative measurements.

A particularly promising application is the derivation of imaging biomarkers. According to the FDA-NIH BEST framework, a biomarker is a defined characteristic measured as an indicator of biological or pathological processes or responses to an intervention (13). Candidate ultrasound biomarkers include tissue thickness, echogenicity, vascularity, follicular morphology, edema, fibrosis, and architectural changes (14–21). These parameters can be assessed serially, although their reproducibility, validity, and clinically meaningful thresholds remain disease- and technique-dependent. In selected dermatoses, sonographic abnormalities may reveal inflammatory activity not apparent clinically, while changes during therapy may provide objective evidence of response (Figure 1) (17–21). Such findings support potential applications in disease stratification, longitudinal monitoring, procedural planning, and treatment assessment, including hidradenitis suppurativa, psoriasis, acne vulgaris, and morphea (19, 20, 22–24).

**Figure 1:**
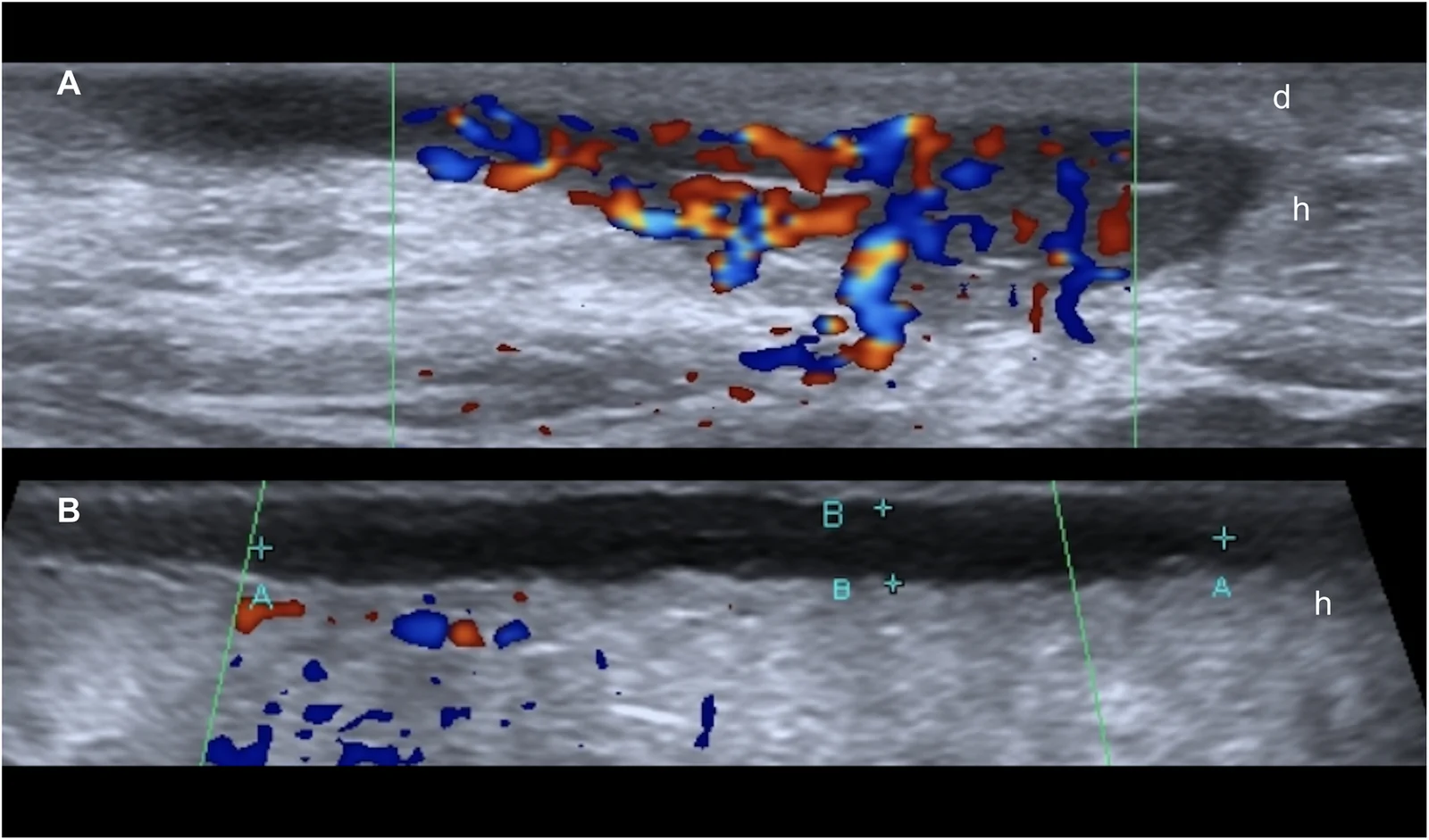
Ultrasound monitoring of therapeutic response in hidradenitis suppurativa. HFUS in a patient with HS. **(A)** Baseline examination before initiation of biologic therapy demonstrates a characteristic fistulous tract with marked Doppler vascularity, consistent with inflammation. **(B)** Follow-up examination after 6 months of treatment shows visibly reduced Doppler vascularity, qualitatively consistent with reduced inflammatory activity. Calipers in panel **(B)** indicate tract dimensions. The images are illustrative and are not intended as a standardized quantitative comparison of tract dimensions or Doppler signal. Images were acquired using a Venue ultrasound system (GE HealthCare, Wauwatosa, WI, USA) with an 18 MHz hockey-stick linear transducer. d, dermis; h, hypodermis. HFUS, high-frequency ultrasound; HS, hidradenitis suppurativa.

This review examines HFUS and UHFUS in inflammatory skin diseases through an imaging biomarker framework, emphasizing shared sonographic features of inflammation, tissue remodeling, and fibrosis and their potential clinical applications.

To inform this narrative review, an extensive literature search was conducted in PubMed and Web of Science from database inception through August 1, 2026. No language restrictions were applied. Searches used combinations of terms including “high-frequency ultrasound,” “ultra-high-frequency ultrasound,” “dermatologic ultrasound,” “skin ultrasound,” “Doppler ultrasound,” “elastography,” “shear-wave elastography,” “imaging biomarker,” “inflammatory skin disease,” “psoriasis,” “atopic dermatitis,” “acne vulgaris,” “hidradenitis suppurativa,” “morphea,” “systemic sclerosis,” “cutaneous lupus erythematosus,” “panniculitis,” “pyoderma gangrenosum,” and “melasma.” Eligible publications included original studies, reviews, consensus statements, and methodological papers addressing ultrasound-derived structural, vascular, or mechanical parameters, disease activity, treatment response, reproducibility, or longitudinal monitoring. Greater weight was given to quantitative, longitudinal, validation, histopathologic-correlation, and consensus-based studies, whereas descriptive reports and small case series were used primarily to illustrate emerging applications. Reference lists of relevant articles were also screened to identify additional pertinent publications. As a narrative review, this synthesis was not designed as a formal systematic review or evidence-grading exercise; heterogeneity in study design, ultrasound platforms, acquisition protocols, and outcome definitions limits direct comparison and precludes quantitative synthesis.

## Ultrasound-derived imaging biomarkers of cutaneous inflammation

HFUS can move cutaneous imaging beyond lesion visualization by generating measurable structural, vascular, and mechanical parameters that may serve as imaging biomarkers of inflammatory activity, tissue remodeling, and repair (19, 21, 25, 26). These parameters can support disease stratification, longitudinal monitoring, and detection of clinically occult abnormalities (Figure 2) (19–21).

**Figure 2:**
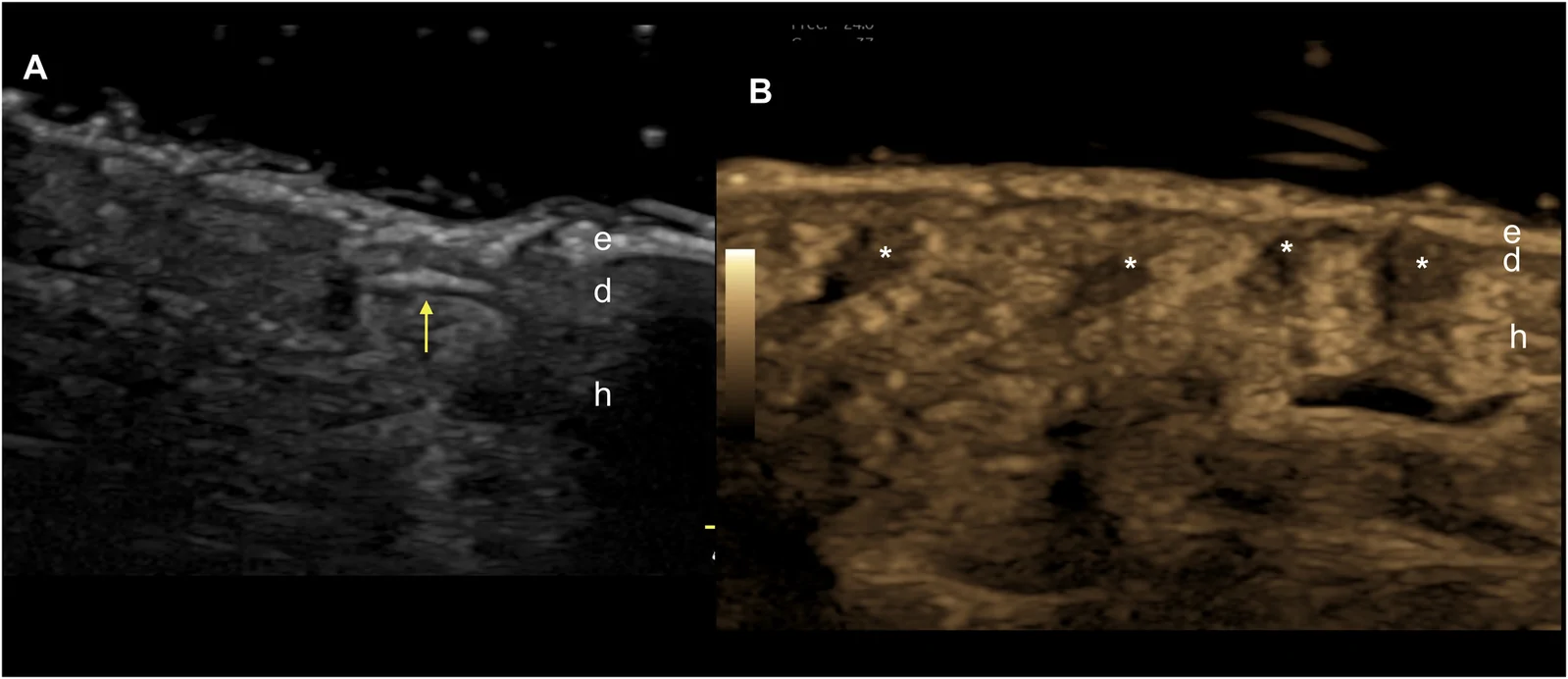
Subclinical ultrasound changes in hidradenitis suppurativa. HFUS demonstrates subclinical changes of HS that are not clinically apparent. **(A)** The yellow arrow indicates a retained hair tract within the dermis, a finding implicated in the development of fistulous tracts. **(B)** Asterisks indicate multiple dilated hair follicles, representing early structural changes of follicular involvement. Panels **(A,B)** represent different skin areas in the same patient and are not longitudinal examinations. Images were acquired using a LOGIQ Totus ultrasound system (GE HealthCare, Wauwatosa, WI, USA) with a 24 MHz hockey-stick transducer. e, epidermis; d, dermis; h, hypodermis. HFUS, high-frequency ultrasound; HS, hidradenitis suppurativa.

For purposes of this review, we distinguish descriptive sonographic findings from candidate imaging biomarkers. Descriptive findings denote observed structural, vascular, or mechanical features, whereas a feature may be considered a candidate imaging biomarker when it is measurable and has evidence linking it to a biological or pathological process, disease state, or response to treatment, consistent with the FDA-NIH BEST framework (13). Where supported by available evidence, we indicate the proposed context of use, including diagnostic, disease-activity/monitoring, treatment-response, prognostic, or predictive applications. The level of validation remains heterogeneous: only selected parameters have demonstrated analytical validity, biological or clinical correlation, responsiveness, reproducibility, or clinical utility. Accordingly, parameters are described as candidate imaging biomarkers unless more substantive validation has been demonstrated. Histopathologic correlations have been reported for selected cutaneous ultrasound features (27), but these relationships should not be generalized across disorders or parameters.

Throughout this review, clinical or biological correlation, detection of clinically occult abnormalities, longitudinal responsiveness, and clinical utility are considered distinct levels of evidence. Clinical utility is reserved for evidence that use of an imaging parameter to guide management improves clinically relevant outcomes; such evidence remains limited for most cutaneous HFUS applications. In the disease-specific sections, analytical validity refers to agreement with an appropriate reference measurement, biological or clinical validity to association with relevant pathological or clinical features, responsiveness to change during longitudinal assessment, and clinical utility to evidence that imaging-informed management improves clinically relevant outcomes. These levels of evidence should not be considered interchangeable. Correlation with established clinical scores alone does not establish incremental clinical value; this requires evidence that HFUS adds information that meaningfully changes diagnosis, staging, treatment selection, monitoring, or prediction of outcomes.

Compared with purely clinical assessment, HFUS offers serial, noninvasive evaluation of the same lesion or anatomical site. This is particularly relevant in chronic inflammatory disease and during biologic therapy, but standardized acquisition and interpretation remain prerequisites for meaningful longitudinal comparison.

### Structural imaging features and candidate biomarkers

Structural ultrasound parameters reflect changes in tissue thickness, echogenicity, architecture, and anatomical interfaces associated with inflammation and remodeling (Table 1). Increased epidermal and/or dermal thickness may reflect inflammatory infiltration, edema, hyperplasia, or extracellular matrix remodeling (20). Frequency-dependent structural patterns have also been described in other immune-mediated dermatoses, reinforcing the need for disease-specific interpretation (28). Skin-layer thickness can be measured serially, although normal values vary with anatomical site and population characteristics (29).

**Table 1:** Major ultrasound-derived imaging features and candidate biomarkers in inflammatory skin disease.

Domain/ultrasound-derived parameter	Disease/context	Modality/representative frequency	Proposed biomarker role	Clinical or histopathological correlate	Longitudinal responsiveness	Validation/reproducibility status
STRUCTURAL Epidermal/dermal thickness (14, 20, 21, 29, 47, 49, 50, 70)	HS; psoriasis; AD; morphea	HFUS/UHFUS; representative studies include 10–22 MHz in HS validation, 20 MHz in morphea, and 70 MHz UHFUS in psoriasis/AD; HS validation: biopsy-matched sites; triplicate measurements averaged	Disease activity; structural burden; longitudinal monitoring	In the HS validation study, sonographic epidermal thickness correlates with histological thickness; the ultrasound measure represented the sonographic entry signal corresponding histologically to psoriasiform epidermal hyperplasia; in morphea, dermal thickness correlates with clinical activity/damage; increased thickness also accompanies inflammatory change in psoriasis and AD.	Treatment-associated reductions reported in psoriasis and AD; responsiveness of individual thickness measures varies by disease and protocol	Analytical validity demonstrated for epidermal thickness in HS; this validation is protocol-specific and should not be generalized to measurement of thin normal epidermis across frequencies or platforms; excellent inter-rater reliability reported for standardized 20-MHz morphea measurements; disease-specific thresholds and cross-device reproducibility remain limited
STRUCTURAL SLEB thickness (46, 47, 49–51, 71)	Psoriasis; AD; CLE	HFUS/UHFUS; 70 MHz VEVO MD used in key longitudinal psoriasis and AD studies	Disease activity; treatment-response monitoring	SLEB reflects superficial dermal edema/inflammation; greater thickness is associated with lesional or active disease and may be detectable in clinically nonlesional AD skin	SLEB thickness decreases during ixekizumab treatment in psoriasis and dupilumab treatment in AD	Candidate biomarker with demonstrated quantitative responsiveness; automated segmentation reported in AD. Standardized thresholds and broad reproducibility data remain limited
STRUCTURAL Dermal echogenicity/architectural change (17, 21, 30, 67–71)	Morphea; connective-tissue panniculitis; CLE	HFUS; 20 MHz DermaLab used in a quantitative morphea protocol	Disease activity vs. fibrosis/remodeling; longitudinal monitoring	Reduced echogenicity may accompany edema/inflammation, whereas increased or heterogeneous echogenicity and architectural distortion may accompany sclerosis/fibrosis; correlations with clinical activity have been reported in morphea	Serial changes have been described, particularly in morphea; longitudinal evidence is less extensive than for SLEB or Doppler vascularity	Clinical correlation and excellent inter-rater reliability reported under a standardized morphea protocol; echogenicity remains acquisition- and device-dependent
STRUCTURAL Follicular abnormalities/pseudocysts (32, 52–55, 61, 62)	HS; acne vulgaris	HFUS/UHFUS; 20 and 50 MHz used in acne studies; multiple frequency ranges used in HS	Disease characterization; severity/staging; detection of clinically occult structural disease	Follicular dilatation, perifollicular changes, pseudocysts, and collections correspond to progressive follicular inflammation/disruption; ultrasound grading has been correlated with clinical severity in acne	Longitudinal data are limited; changes may accompany treatment, but formal responsiveness criteria are not established	Primarily descriptive findings/candidate biomarkers. Clinical-ultrasound associations have been reported, but formal analytical validation is limited
STRUCTURAL Tunnel/fistulous-tract diameter and morphology (14, 32, 35–39, 61–66)	HS; exploratory evidence in acne	HFUS; representative HS validation and longitudinal studies used approximately 10–22 MHz	Structural disease burden; staging; treatment/procedural planning; treatment-response monitoring	In HS, sonographic tunnel diameter correlates with histological tunnel diameter; morphology and surrounding fibrosis may inform management	Tunnel dimensions and vascular features may change during biologic therapy; longitudinal responsiveness has been reported in HS	Management impact for staging/planning is supported by consensus and observational literature; evidence that ultrasound-guided decisions improve patient outcomes remains insufficient
VASCULAR Color/power Doppler vascularity (14, 20, 33–39, 46, 47, 50, 59, 69)	HS; psoriasis; AD; morphea	Color/power Doppler with HFUS/UHFUS; acquisition depends on probe, Doppler gain, PRF, wall filter, and pressure HS validation: power Doppler intensity graded semiquantitatively on a 1–3 scale.	Disease activity; treatment-response monitoring; potential early-response marker	In HS, power Doppler intensity correlates with dermal inflammatory-cell infiltrates and several clinical activity measures; increased vascularity also accompanies active psoriasis, AD, and morphea	Reductions in vascularity reported during systemic/biologic therapy in HS, psoriasis, and AD	Analytical and clinical correlation demonstrated for power Doppler intensity in HS. Responsiveness has been reported, but evidence that Doppler-guided decisions improve patient outcomes remains limited; reproducibility is protocol- and device-dependent
MECHANICAL Skin stiffness/shear-wave elastography (40–42)	Systemic sclerosis; emerging relevance to fibrosing dermatoses	SWE combined with HFUS; e.g., 24 MHz linear probe on Aplio i900 in a representative systemic-sclerosis study	Fibrosis/mechanical tissue assessment; potential longitudinal monitoring	Stiffness has been investigated against clinical, histological, and molecular measures of fibrosis/remodeling, but correlations are not uniformly consistent	Longitudinal treatment-response evidence remains limited	Candidate mechanical biomarker. Values vary with anatomical site, acquisition technique, equipment, and disease stage; broader standardization and validation are required

AD, atopic dermatitis; CLE, cutaneous lupus erythematosus; HFUS, high-frequency ultrasound; HS, hidradenitis suppurativa; PRF, pulse-repetition frequency; SLEB, subepidermal low-echogenic band; SWE, shear-wave elastography; UHFUS, ultra-high-frequency ultrasound.

Frequencies and platforms are representative examples from key quantitative studies and are not intended to imply interchangeability across systems. Validation status summarizes available evidence for analytical validity, clinical or histopathological correlation, longitudinal responsiveness, and reproducibility; it is not a formal evidence-grading system. Most parameters remain candidate imaging biomarkers. Reference numbers correspond to the manuscript bibliography. Underlying evidence ranges from descriptive reports and small case series to longitudinal, validation, and consensus studies; Table 1 represents a narrative synthesis rather than a formal study-level evidence hierarchy.

Altered echogenicity is another important structural parameter. Acute inflammation often increases tissue water content and reduces dermal echogenicity. With resolution, echogenicity and tissue architecture may normalize; by contrast, chronic collagen deposition and sclerosis may produce increased or heterogeneous echogenicity with architectural distortion (30). Echogenicity can therefore help characterize inflammatory vs. fibrotic predominance, but it is not disease-specific.

Blurring or loss of the normal dermo-hypodermal interface may indicate deeper tissue involvement, particularly in disorders such as morphea (21). Other dermatologic conditions may show overlapping structural abnormalities, including altered echogenicity and subepidermal changes, underscoring the need to interpret individual ultrasound findings in clinical context rather than as disease-specific biomarkers (31). Follicular inflammatory disorders show additional structural abnormalities, including follicular dilatation, perifollicular hypoechogenicity, pseudocysts, fluid collections, and tunnels. In hidradenitis suppurativa, these findings can represent progressive follicular disruption and interconnected tract formation that is directly visualized by HFUS (Figure 3) (32). Progressive fibrotic remodeling may additionally appear as heterogeneous hyperechoic bands that distort or replace normal dermal architecture.

**Figure 3:**
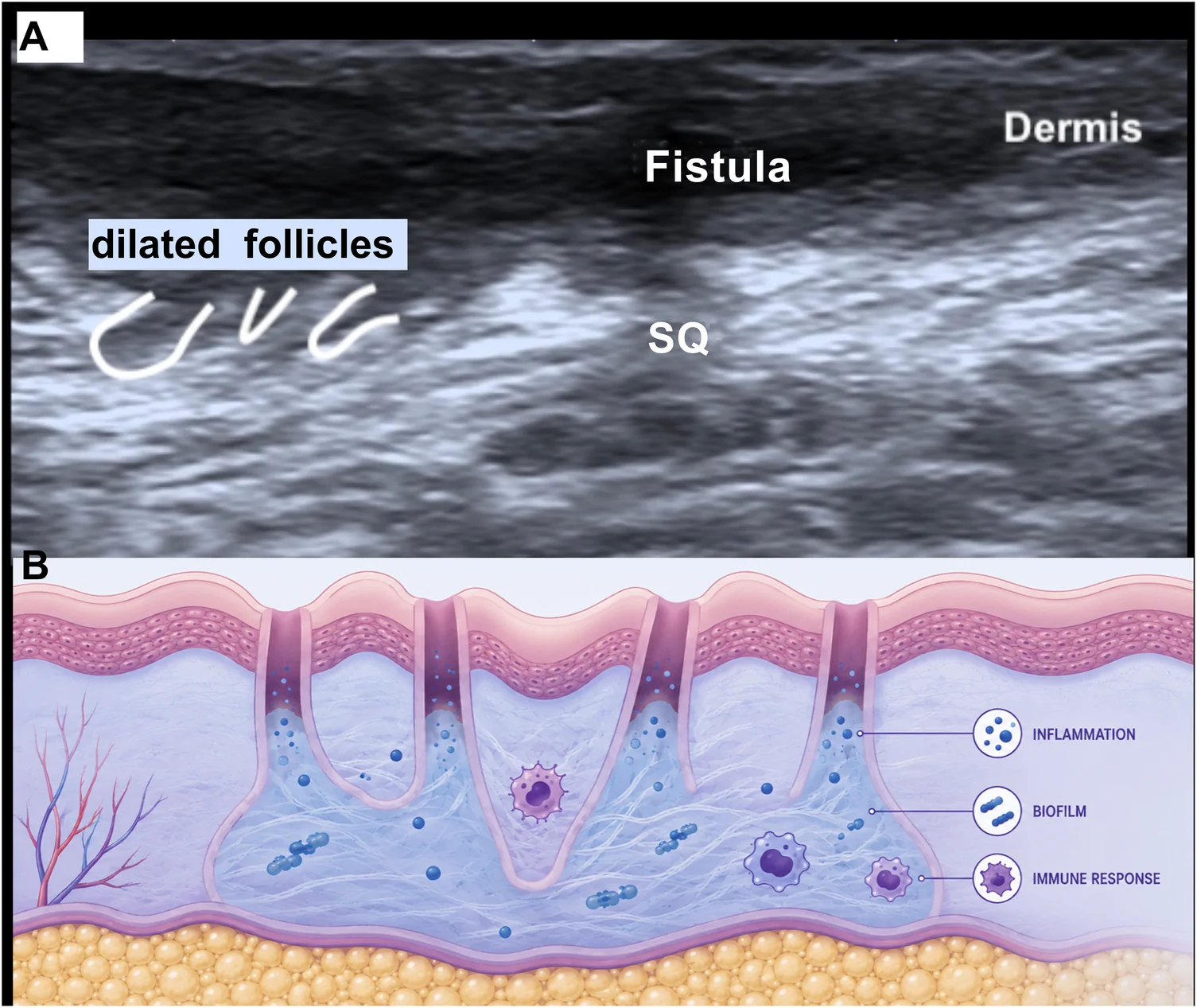
Follicular alterations associated with fistula formation in hidradenitis suppurativa. **(A)** HFUS demonstrates a fistulous tract in HS, with multiple dilated hair follicles at its base (outlined in white). These follicular abnormalities represent early structural changes implicated in the development and interconnection of fistulous tracts. **(B)** Schematic representation of follicular inflammation and associated pathogenic processes contributing to fistula formation in HS. The schematic in panel **(B)** was created by the authors. The HFUS image in panel **(A)** was acquired using a Venue ultrasound system (GE HealthCare, Wauwatosa, WI, USA) with an 18 MHz hockey-stick linear transducer. HFUS, high-frequency ultrasound; HS, hidradenitis suppurativa; SQ, subcutaneous tissue.

### Vascular imaging features and candidate biomarkers

Inflammation is associated with vasodilation, endothelial activation, angiogenesis, and increased tissue perfusion. Accordingly, Doppler assessment provides a functional complement to grayscale morphology (33).

Color and power Doppler can demonstrate increased vascular signal within inflamed tissue, while newer microvascular techniques improve sensitivity to slow flow in small-caliber vessels (34). In several inflammatory disorders, Doppler signal correlates with clinical or sonographic activity; longitudinal studies, particularly in hidradenitis suppurativa, have shown reductions in vascularity during systemic or biologic therapy, sometimes before complete structural resolution (36–39). The ultrasonographic “railway sign” in HS tunnels has also been reported as an independent predictor of adalimumab failure, illustrating a potential predictive imaging-biomarker application (35). Serial Doppler assessment may therefore serve as a candidate treatment-response biomarker, provided acquisition settings are standardized; whether Doppler-guided treatment modification improves clinical outcomes remains to be established. Doppler assessment may be qualitative, semiquantitative, or quantitative, and these approaches are not interchangeable. Although semiquantitative power-Doppler grading has demonstrated analytical and clinical validity in HS under a defined protocol, validated disease-specific thresholds remain limited. Treatment-associated reduction in Doppler signal should therefore generally be interpreted as longitudinal responsiveness and support for a candidate treatment-response biomarker rather than a validated endpoint, unless additional validation has been demonstrated.

### Mechanical imaging features and candidate biomarkers

Elastography adds an emerging measure of tissue mechanical properties, particularly stiffness related to fibrosis and extracellular matrix remodeling. In systemic sclerosis, shear-wave elastography has been evaluated alongside skin thickness and echogenicity using a 24 MHz linear transducer on an Aplio i900 system (Canon Medical Systems, Otawara, Japan), with measurements acquired without skin compression by the probe (40). However, systematic and validation studies show that values are influenced by anatomical site, acquisition technique, equipment, and disease stage, and correlations with clinical measures are not uniformly consistent (41, 42). Elastography-derived parameters should therefore be regarded as candidate mechanical biomarkers rather than established measures of inflammatory activity or fibrosis, pending further standardization and validation (41). Comparability also depends on probe pressure, patient positioning, region-of-interest size and depth, number of repeated measurements, and reporting units; these factors should therefore be standardized and explicitly reported. Beyond elastography, ultrasound-measured skin thickness in systemic sclerosis has demonstrated good reliability and convergent validity against the modified Rodnan skin score and histological thickness, although acquisition and analysis methods remain heterogeneous (41).

## Clinical applications of HFUS in inflammatory skin diseases

HFUS identifies recurring structural and vascular patterns across inflammatory dermatoses while also revealing disease-specific morphology. Its clinical value lies in complementing examination with objective information on lesion depth, inflammatory activity, tissue damage, and response over time. The principal disease sections were selected because they have comparatively more developed evidence supporting quantitative assessment, longitudinal monitoring, clinical correlation, treatment-response evaluation, or management impact; conditions supported mainly by limited or preliminary studies are discussed under *Other Emerging Applications*. The clinical examples shown in the figures are illustrative and should not be interpreted as independent evidence of validated diagnostic, prognostic, or treatment-guiding performance.

### Psoriasis

Nail psoriasis is among the best-established dermatologic applications of ultrasound. Sonographic findings include thickening of the nail matrix and nail bed, irregularity of the ventral nail plate, loss of normal trilaminar architecture, and hyperechoic foci with posterior acoustic shadowing (43–45). Some structural abnormalities may precede overt nail deformity, supporting ultrasound as a complementary tool when clinical findings are equivocal (Figure 4A,B).

**Figure 4:**
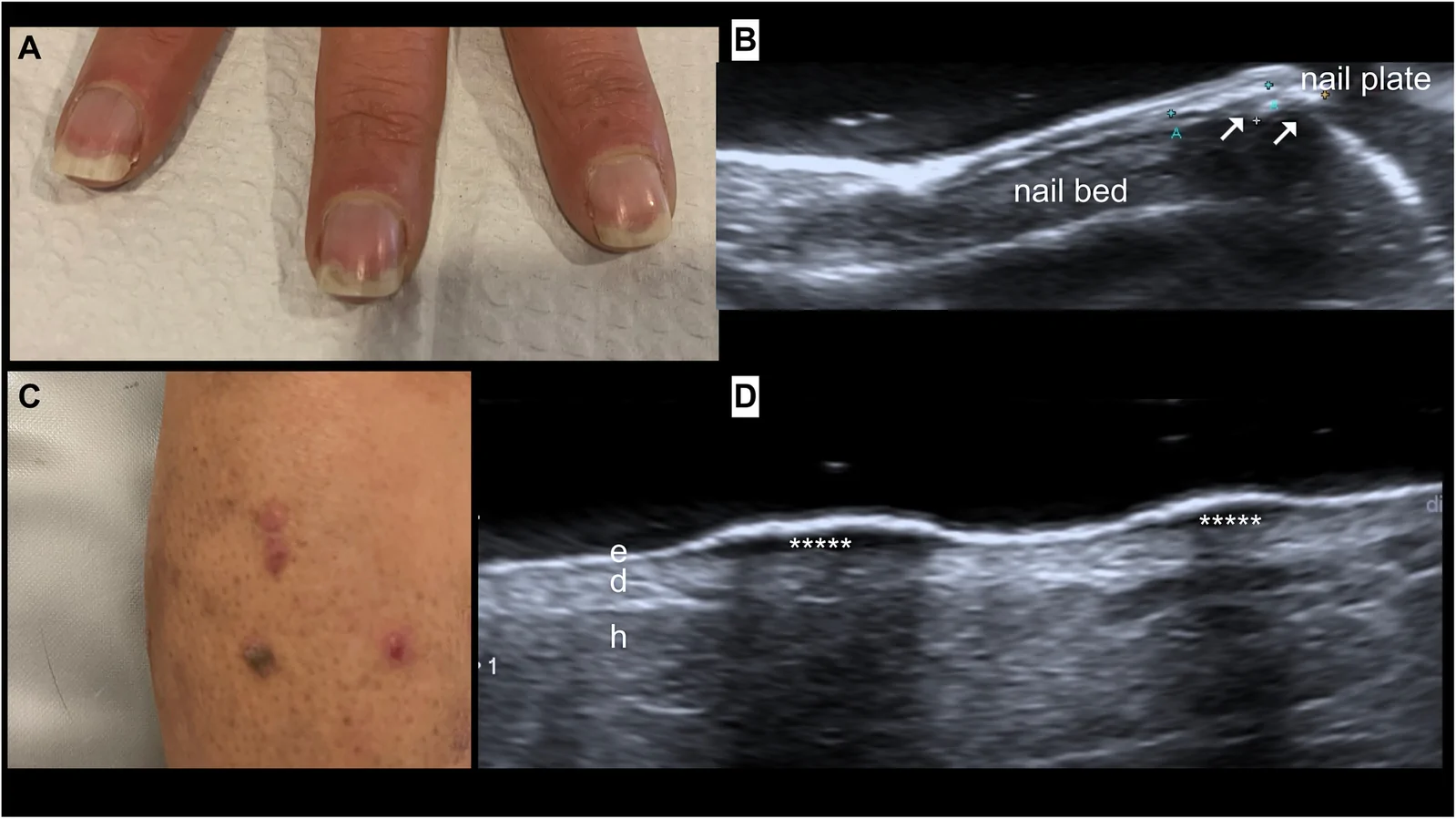
Clinical and ultrasound findings in nail and plaque psoriasis. **(A)** Clinical image showing nail changes consistent with psoriasis, including onycholysis. **(B)** HFUS examination of the nail. White arrows indicate hyperechoic foci with posterior acoustic shadowing involving the ventral nail plate, a characteristic sonographic feature of nail psoriasis. **(C)** Clinical image showing psoriatic plaques on the leg. **(D)** HFUS of a psoriatic plaque. Asterisks indicate a superficial horizontal hypoechoic band consistent with SLEB corresponding to superficial dermal edema and inflammation; vertically oriented acoustic shadowing related to increased keratin content in the hyperplastic psoriatic epidermis is also present. The HFUS images in panels **(B,D)** were acquired using a Venue ultrasound system (GE HealthCare, Wauwatosa, WI, USA) with an 18 MHz hockey-stick linear transducer. These 18-MHz images are illustrative and are not intended to imply quantitative comparability with the 70 MHz UHFUS measurements discussed in the text. e, epidermis; d, dermis; h, hypodermis. HFUS, high-frequency ultrasound.

Psoriatic plaques may show epidermal thickening and a subepidermal low-echogenic band (SLEB) consistent with superficial dermal edema and inflammation, together with increased Doppler vascularity (15, 46). In a longitudinal UHFUS study using a 70-MHz VEVO MD system, reductions in SLEB thickness and vascular signal were demonstrated during ixekizumab treatment, demonstrating longitudinal responsiveness and supporting their candidacy as treatment-response biomarkers (Figure 4C,D) (47). Thus, SLEB thickness and vascular signal demonstrate longitudinal responsiveness, but standardized thresholds, cross-platform reproducibility, and incremental clinical utility remain incompletely established.

Ultrasound may also identify associated enthesitis, tenosynovitis, or other features of psoriatic arthritis when clinically relevant (43, 48). These musculoskeletal applications are complementary but extend beyond the principal cutaneous focus of this review.

### Atopic dermatitis

Atopic dermatitis (AD) is another inflammatory dermatosis in which HFUS may provide candidate imaging biomarkers of disease activity. A characteristic, but not disease-specific, finding is SLEB, reflecting increased water content and inflammation in the superficial dermis. SLEB thickness is greater in lesional skin and may also be present in clinically nonlesional skin, suggesting subclinical involvement. Reduced dermal echogenicity and increased epidermal thickness provide additional structural parameters (49).

Using a VEVO MD system with a 70-MHz probe, longitudinal UHFUS assessment during dupilumab therapy demonstrated reductions in SLEB thickness, epidermal thickness, and vascular signal in lesional skin (50). Automated HFUS analysis has also shown promising performance for SLEB segmentation and AD severity classification, illustrating the potential for more standardized image interpretation (51). These findings support longitudinal responsiveness, while disease-specific thresholds, cross-platform reproducibility, and clinical utility remain incompletely established.

### Acne vulgaris

Clinical examination may underestimate the depth and extent of inflammatory acne lesions, particularly deep nodules, pseudocysts, abscesses, and fistulous tracts (52). HFUS can therefore complement surface inspection when lesion depth or severity is uncertain. HFUS can demonstrate deep folliculitis, inflammatory nodules, pseudocysts, dermal abscesses, fistulous tracts, and calcifications (Figure 5) (52, 53). These findings may reveal inflammatory extension beyond clinically visible papules or pustules.

**Figure 5:**
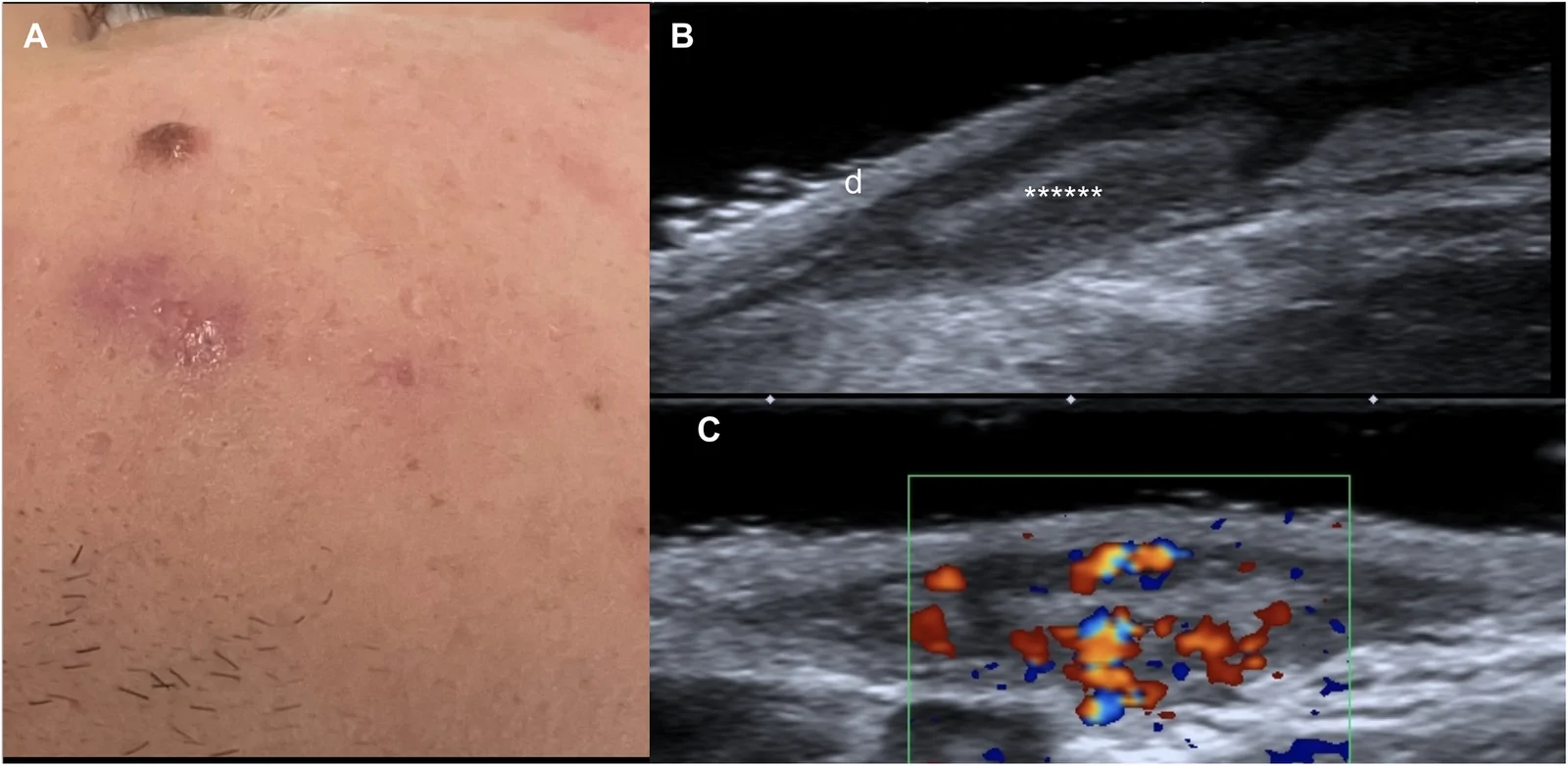
Ultrasound-based reassessment of acne severity and therapeutic management. **(A)** Clinical image of acne vulgaris with inflammatory lesions that had responded inadequately to topical therapy. **(B)** HFUS demonstrates that a lesion clinically interpreted as a nodule corresponds to a dermohypodermal fistulous tract (asterisks), revealing deeper structural involvement than apparent on clinical examination. **(C)** Doppler examination demonstrates marked vascularity within and around the fistulous tract, consistent with active inflammation. These findings support reassessment of disease severity and consideration of systemic therapy. This case is illustrative and does not establish validated treatment-guiding performance. The HFUS images in panels **(B,C)** were acquired using a Venue ultrasound system (GE HealthCare, Wauwatosa, WI, USA) with an 18 MHz hockey-stick linear transducer. d, dermis. HFUS, high-frequency ultrasound.

Beyond lesion characterization, sonographic scoring systems have been proposed. The Sonographic Scoring of Acne (SOS-Acne) distinguishes active features such as pseudocysts and fistulous tracts from chronic sequelae such as calcifications and scars (52–55). Integration of HFUS with the Global Acne Grading System has also been investigated for prediction of treatment response (56). This distinction illustrates the potential of ultrasound to separate current inflammatory activity from irreversible structural damage. Current evidence remains predominantly descriptive and associative, and broader validation, reproducibility, and incremental clinical value of proposed ultrasound scores remain limited.

### Hidradenitis suppurativa

Hidradenitis suppurativa (HS) is among the inflammatory skin diseases in which HFUS has had the greatest impact on staging, monitoring, and procedural planning (57–60). In HS, ultrasound findings may directly influence clinical staging and management by revealing clinically occult tunnels, collections, and disease extension that are not apparent on physical examination (Figure 6). This represents a more advanced level of clinical application than simple correlation or treatment responsiveness, although prospective evidence that predefined ultrasound-guided management algorithms improve long-term outcomes remains limited. HS evolves through follicular occlusion and dilatation, rupture, perifollicular inflammation, collections, and tunnel formation (61). Correlations between UHFUS and histopathological features further support the biological relevance of the sonographic findings (62). Among the parameters reviewed, sonographic epidermal thickness and dermal tunnel diameter have demonstrated analytical validity against histopathology, while power Doppler intensity has shown both analytical and clinical validity in HS (14).

**Figure 6:**
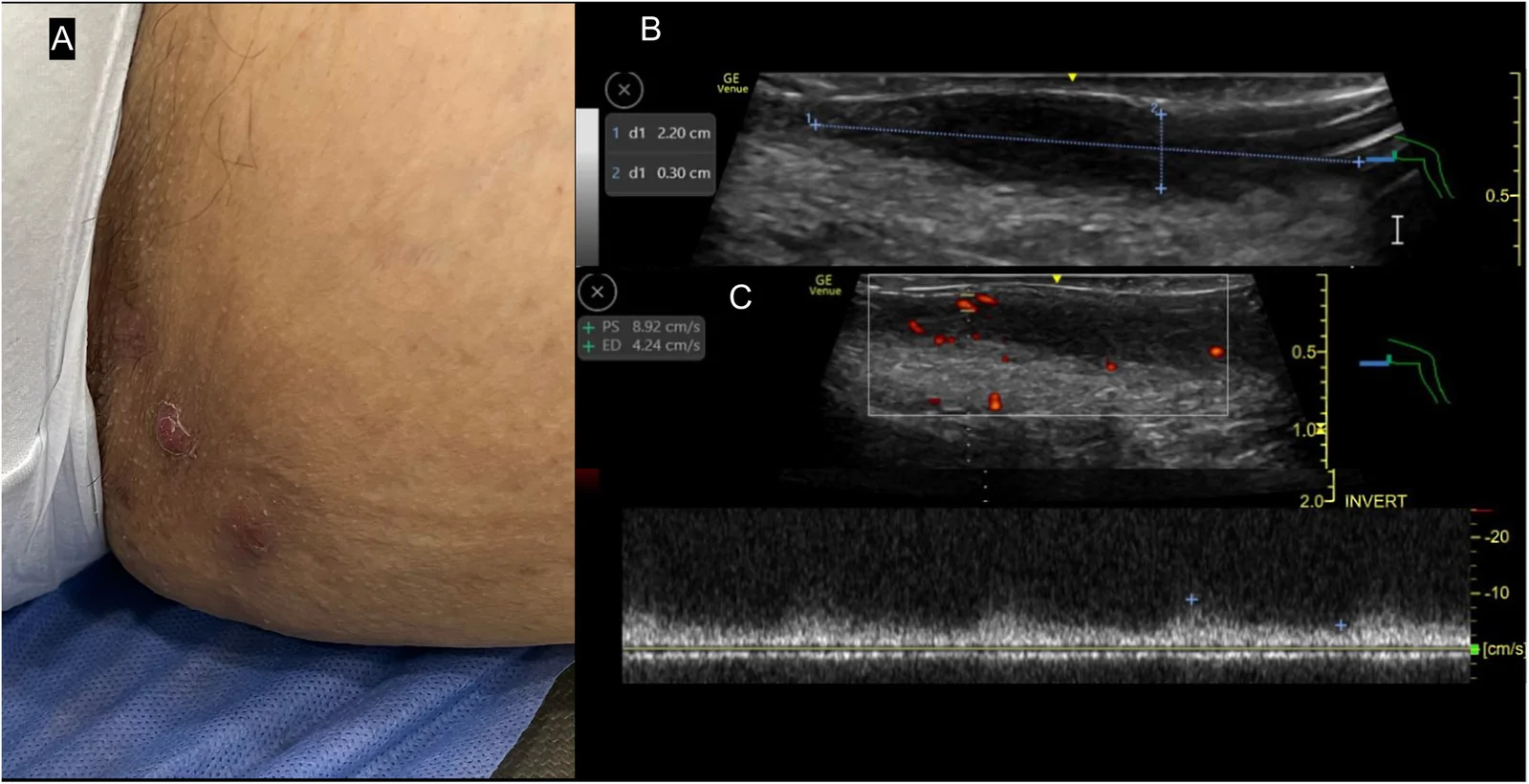
Ultrasound-based reassessment of disease staging and therapeutic planning in hidradenitis suppurativa. **(A)** Clinical image of a patient with HS showing lesions in the proximal medial thigh that were clinically interpreted as nodules, consistent with Hurley stage I disease. **(B)** HFUS demonstrates that the clinically identified lesion corresponds to a dermohypodermal fistulous tract, measuring approximately 2.20 cm in length and 0.30 cm in thickness. **(C)** Doppler examination demonstrates increased vascularity within the tract, with a peak systolic velocity of 8.9 cm/s, consistent with active inflammation. Detection of the fistulous tract enabled reassessment of the disease as Hurley stage II and modification of therapeutic planning. This case illustrates potential management impact but does not establish improved outcomes from ultrasound-guided management. The HFUS images in panels **(B,C)** were acquired using a Venue ultrasound system (GE HealthCare, Wauwatosa, WI, USA) with an 18 MHz hockey-stick linear transducer. HFUS, high-frequency ultrasound; HS, hidradenitis suppurativa.

Characteristic findings include widened hair follicles, dermal thickening, pseudocysts, inflammatory collections, tunnels, surrounding edema, and Doppler hypervascularity. Ultrasound-based classifications, including the modified Sonographic Score of HS (mSOS-HS), incorporate objective findings into severity and activity assessment, while standardized reporting facilitates longitudinal comparison (19, 63, 64). Tunnel morphology and surrounding fibrosis may further inform therapeutic planning, including consideration of medical vs. surgical approaches (65, 66). Thus, HS has comparatively stronger evidence for analytical/clinical validity and management impact, but prospective evidence that ultrasound-guided decisions improve long-term outcomes remains insufficient.

### Morphea

Morphea is particularly suited to ultrasound assessment because inflammatory activity and progressive fibrosis may coexist within the same lesion (67, 68). Active lesions may demonstrate dermal thickening, decreased echogenicity, increased vascularity, subcutaneous edema, and loss of the normal dermo-hypodermal interface. As fibrosis progresses, vascularity tends to diminish while echogenicity increases with collagen deposition and tissue sclerosis (69). In a quantitative study using 20 MHz HFUS (DermaLab System, Cortex Technology, Hadsund, Denmark), lesional dermal thickness and echogenicity were compared with site-matched unaffected skin, illustrating the importance of standardized acquisition frequency and reference-site selection for quantitative assessment (70).

Distinguishing active inflammation from established fibrosis has important therapeutic implications (Figure 7). Persistent Doppler activity in a clinically quiescent lesion may suggest residual inflammatory activity and prompt reassessment, whereas low vascularity together with established fibrosis may support inactive disease (21, 67, 69, 70). Serial ultrasound can therefore complement clinical evaluation when disease activity is uncertain. Quantitative assessment has shown clinical correlation and reproducibility under standardized protocols, but disease-specific thresholds and inter-device transferability remain limited.

**Figure 7:**
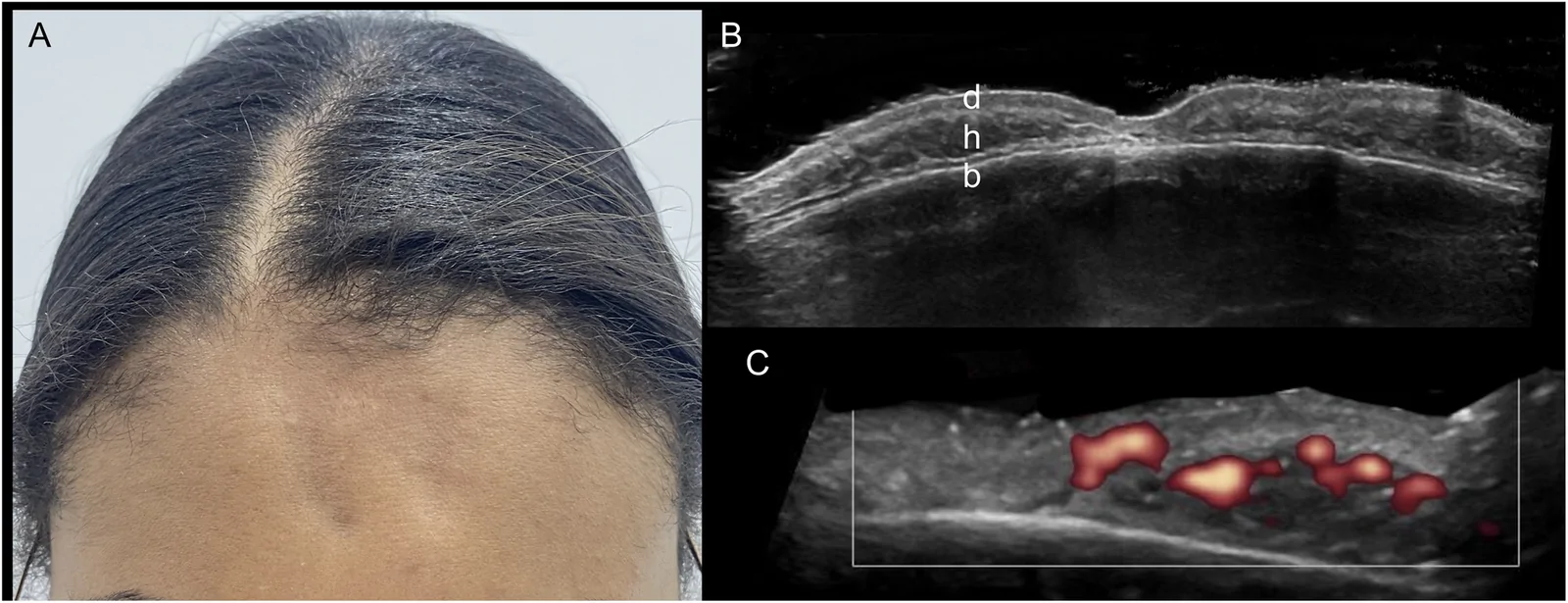
Ultrasound detection of persistent inflammatory activity in clinically atrophic morphea. **(A)** Clinical image of frontal morphea with marked cutaneous atrophy. **(B)** HFUS demonstrates pronounced tissue atrophy, predominantly involving the hypodermis. **(C)** Doppler examination shows increased hypodermal vascularity, consistent with persistent inflammatory activity despite the clinically atrophic appearance. This case is illustrative and does not establish a validated activity threshold. The HFUS images in panels **(B,C**) were acquired using a Venue ultrasound system (GE HealthCare, Wauwatosa, WI, USA) with an 18 MHz hockey-stick linear transducer. d, dermis; h, hypodermis; b, bone. HFUS, high-frequency ultrasound.

### Other emerging applications

HFUS is also being explored in less extensively studied inflammatory and inflammation-associated dermatoses for which the evidence remains limited. In connective-tissue panniculitis, changes in dermal and hypodermal echogenicity and Doppler vascularity may help distinguish active inflammation from predominantly residual or chronic changes and may complement therapeutic decision-making (17). In cutaneous lupus erythematosus, increased skin thickness and the presence and thickness of SLEB have been associated with active disease, whereas increased echogenicity may accompany more chronic or inactive changes (71). Melasma is primarily a pigmentary disorder with a recognized vascular component and, in some cases, chronic inflammatory changes; accordingly, its inclusion here represents an exploratory inflammation-associated application of HFUS rather than an application in a primarily inflammatory dermatosis. A small prospective case series reported SLEB, follicular dilatation, and increased vascularity at baseline, with reduction in SLEB and improved echogenicity after Q-switched Nd:YAG laser treatment, suggesting a potential role for HFUS in characterizing subclinical inflammatory changes and monitoring treatment response (31). Accordingly, the evidentiary level for this application remains preliminary.

Preliminary UHFUS studies in pyoderma gangrenosum have identified structural and vascular differences between inflammatory and noninflammatory phases, suggesting potential applications in disease staging and treatment monitoring (9). These applications remain promising but require further validation before ultrasound-derived parameters can be considered established biomarkers in routine practice.

## Future directions: toward precision dermatology

HFUS is evolving from primarily morphological imaging toward quantitative and functional assessment of cutaneous disease (72, 73). Higher-frequency systems improve visualization of superficial structures and have expanded *in vivo* study of the epidermis, superficial dermis, hair follicles, nail unit, and appendages (5, 74, 75). The clinical value of these advances will depend less on maximal spatial resolution alone than on whether measurable features improve disease classification, monitoring, or management.

In selected disorders, HFUS may detect structural or vascular abnormalities before they are clinically obvious, offering a potential window for earlier recognition of disease activity or treatment response (46, 70, 76, 77). However, evidence that earlier sonographic detection improves long-term outcomes remains limited and requires prospective validation.

Quantitative image analysis is a major area of development. Automated measurement of skin thickness, lesion volume, vascularity, echogenicity, and tissue heterogeneity could improve reproducibility and facilitate multicenter studies. Artificial intelligence (AI) may further support segmentation, feature extraction, and longitudinal comparison. Direct evidence in inflammatory dermatology remains limited, although automated HFUS analysis in AD has shown promising performance; proof-of-concept work in other dermatologic ultrasound applications demonstrates broader technical feasibility (51, 78–80). Future multimodal models could integrate ultrasound-derived features with clinical and molecular data, but prediction of disease phenotype or treatment response remains investigational. Clinical translation will require larger multicenter datasets, external validation, evaluation across operators, devices, and populations, and explicit assessment of generalizability and dataset bias.

Repeated, noninvasive bedside imaging is particularly attractive as biologic and targeted therapies expand (15, 46). Future trials could incorporate selected ultrasound parameters as exploratory or secondary outcomes to determine responsiveness, clinically meaningful thresholds, and added value beyond established clinical measures.

Quantitative ultrasound parameters are sensitive to acquisition conditions. Structural measures may vary with anatomical site, transducer frequency and bandwidth, ultrasound platform, gain/TGC, dynamic range, probe pressure, and reference-skin selection, while Doppler vascularity is particularly influenced by equipment and settings such as Doppler mode, gain, pulse-repetition frequency, wall filter, and probe pressure. Environmental and skin-surface temperature may also affect ultrasound backscatter and echogenicity (81). A recent multicenter study of healthy skin demonstrated differences in vascular detection according to probe frequency and equipment despite standardized Doppler acquisition (33). Accordingly, longitudinal assessment should ideally use consistent anatomical sites, systems, transducers, acquisition settings, probe pressure, environmental conditions, and reference strategies, following established dermatologic-ultrasound guidance where applicable (82, 83).

Available reproducibility data are encouraging but heterogeneous. Inter- and intra-observer agreement has been demonstrated for selected dermatologic ultrasound findings, but reproducibility remains dependent on the parameter, disease, acquisition protocol, and operator experience; evidence for inter-device reproducibility is considerably more limited (18, 82–84). Quantitative thresholds derived from one system or protocol should therefore not automatically be assumed to transfer across platforms. Cross-platform validity should therefore be assumed only when specifically demonstrated; most quantitative thresholds remain protocol-, frequency-, and device-specific. Representative observer-agreement data include dermatologic ultrasound assessment of panniculitis and standardized 20 MHz morphea measurements of dermal thickness and echogenicity; comparable inter-device data remain sparse (18, 70).

Remaining barriers include limited normative reference data, operator dependence, and incomplete validation of quantitative thresholds (82–84). Training and consensus-based protocols are therefore essential, as are multicenter studies demonstrating reproducibility and clinically meaningful change. Future reference ranges will require multicenter normative datasets accounting, where relevant, for anatomical site, age, sex, population characteristics, device/frequency, and acquisition protocol.

HFUS may increasingly become one component of multimodal assessment alongside clinical examination, dermoscopy, laboratory biomarkers, histopathology, and molecular profiling (72, 73, 85). Its role should be complementary, providing real-time structural and vascular information where that information demonstrably improves phenotyping, monitoring, or treatment decisions.

## Conclusions

HFUS provides noninvasive assessment of structural and vascular changes associated with inflammatory skin disease, while elastography offers an emerging measure of tissue mechanical properties. Ultrasound-derived parameters may complement clinical evaluation by identifying subclinical abnormalities and providing serial information on disease activity, tissue remodeling, and treatment response. Broader clinical translation will require standardized acquisition protocols, validated biomarkers and thresholds, reproducibility across operators and centers, and prospective evidence demonstrating an impact on therapeutic decision-making and outcomes. Integration with clinical, dermoscopic, histopathological, laboratory, and molecular data may further strengthen its role in multimodal disease assessment. The level of clinical validation is heterogeneous, with some applications remaining exploratory and others, particularly in HS, already informing disease staging and therapeutic planning. With continued validation and standardization, HFUS-derived candidate biomarkers may contribute to precision dermatology and future treat-to-target strategies, but this will require prospective demonstration that imaging-guided management improves clinically relevant outcomes.
